# Morphology of the Sella Turcica: A Meta-Analysis Based on the Results of 18,364 Patients

**DOI:** 10.3390/brainsci13081208

**Published:** 2023-08-15

**Authors:** Tomasz Iskra, Bartłomiej Stachera, Kamil Możdżeń, Agnieszka Murawska, Patryk Ostrowski, Michał Bonczar, Iwona Gregorczyk-Maga, Jerzy Walocha, Mateusz Koziej, Grzegorz Wysiadecki, Krzysztof Balawender, Andrzej Żytkowski

**Affiliations:** 1 Department of Anatomy, Jagiellonian University Medical College Cracow, 33-332 Kraków, Poland; tomasz.iskra@uj.edu.pl (T.I.); bartek.s2304@gmail.com (B.S.); kamil.mozdzen@student.uj.edu.pl (K.M.); aga.murawska@student.uj.edu.pl (A.M.); ostrowskipatryk0@gmail.com (P.O.); michalbonczar01@gmail.com (M.B.); j.walocha@uj.edu.pl (J.W.); mateusz.koziej@gmail.com (M.K.); 2 Youthoria—Youth Research Organization, 33-332 Kraków, Poland; 3 Faculty of Medicine, Institute of Dentistry, Jagiellonian University Medical College, 31-155 Krakow, Poland; iwona.gregorczyk-maga@uj.edu.pl; 4 Department of Normal and Clinical Anatomy, Chair of Anatomy and Histology, Medical University of Lodz, 90-752 Łódź, Poland; 5 Department of Normal and Clinical Anatomy, Institute of Medical Sciences, Medical College, Rzeszow University, 35-315 Rzeszów, Poland; balawender82@gmail.com; 6 Norbert Barlicki Memorial Teaching Hospital No. 1, Medical University of Lodz, 90-001 Łódź, Poland; andrzej.zytkowski.anat@gmail.com

**Keywords:** morphometry, neuroanatomy, pituitary gland, pituitary fossa, sella turcica, skull base, sphenoid bone

## Abstract

Background: This meta-analysis aimed to present data on the sella turcica (ST) morphology and variations. Furthermore, a detailed morphometric analysis of the ST was conducted. Methods: Major online databases such as PubMed, Scopus, Embase, Web of Science, and the Cochrane Library were searched through. The overall search process was conducted in three stages. Results: This meta-analysis was based on the results of 18,364 patients and demonstrates the most up-to-date and relevant data regarding the morphology of the ST in the available literature. Four classification methods of the ST shape can be distinguished, in which the most commonly occurring variants are the normal ST (55.56%), the U-shaped ST (73.58%), the circular type of ST (42.29%), and non-bridging ST (55.64%). The overall midpoint height of the ST was 6.59 mm (SE = 0.13). The overall length of the ST was 9.06 mm (SE = 0.15). The overall volume of the ST was 845.80 mm^3^ (SE = 288.92). Four main classification methods of ST morphology can be distinguished in the available literature. Various morphometric characteristics of the ST may be applied in clinical practice to evaluate its shape, dimensions, and normal or pathological variants.

## 1. Introduction

**Figure 1:**
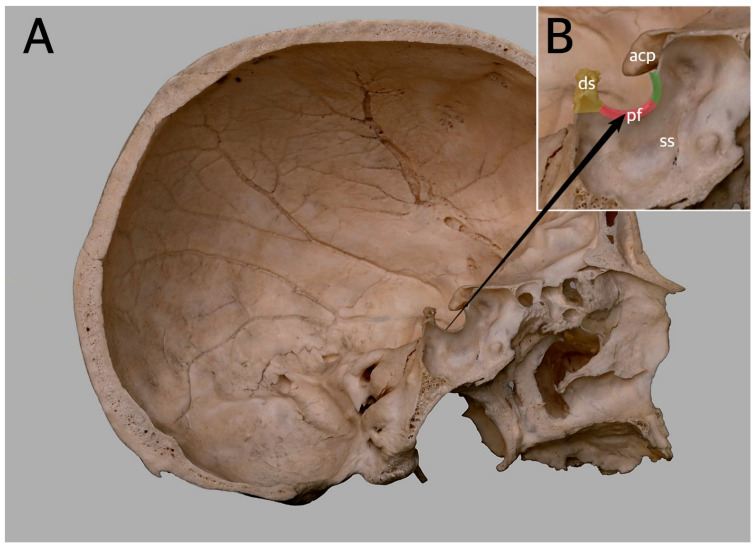
Sagittal section of the macerated adult human skull. (**A**) General view. (**B**) Enlargement of the sella turcica, showing its boundaries. The anterior wall of the sella is marked pale green. The pituitary fossa (pf) on the sella floor is marked pale pink. The dorsum sellae (ds), forming the posterior boundary, is marked pale yellow. acp—anterior clinoid process; ss—sphenoidal sinus.

The sella turcica (ST) is a saddle-shaped depression on the superior part of the sphenoid bone (Figure 1) [1]. It is located in the middle cranial fossa, and it is bounded posteriorly by the dorsum sellae (DS) and anteriorly by the tuberculum sellae (TS) [2,3,4,5,6,7,8]. The hypophyseal fossa is the most inferior part of ST (Figure 1), and it contains the hypophysis, also known as the pituitary gland (PG). The PG consists of three lobes: the anterior lobe (adenohypophysis), the intermediate lobe (a thin layer of cells), and the posterior lobe (neurohypophysis) [9,10,11]. It is one of the endocrine system’s key regulatory structures since it secretes numerous hormones that influence other endocrine glands [12].

Due to anatomical relationships, the alteration in ST shape and dimensions, e.g., ST bridging, can be clinically significant. For example, deviations in the ST morphology are mostly seen in patients with various congenital disorders such as Down syndrome, Seckel syndrome, Williams syndrome, and Axenfeld–Rieger syndrome. Furthermore, this causation can be reversed, and PG ailments can cause ST malformations [12,13,14]. It can be a significant factor to consider, as studies have shown that calcification of the ST ceases after full PG development. Therefore, pathologies causing PG enlargements, such as adenomas or cysts, or shrinkage, e.g., primary hypopituitarism or growth hormone secretion deficiency, may potentially impact the ST’s definitive shape [12,14].

ST and PG have two embryological origins, with the posterior part of each structure derived from the paraxial mesoderm, which is highly dependent on notochordal induction, and the anterior part originating from the neural crest cells. This factor, along with the different embryogenic processes, explains the observation that anomalies of the anterior wall of the ST appear to be associated with changes in the frontonasal area and body axis defects. In contrast, anomalies of the posterior wall of the ST are linked to changes in the cerebrum [12]. Hence, the ST size and shape are closely related to PG morphology. 

Normality in anatomy can be considered a type of arrangement based on numerous repeated observations [15]. Various studies were conducted to assess ST morphology, yet most included a small number of participants and obtained different results. Thus, the main objective of the present meta-analysis was to provide reliable and objective data on the structure of ST in various patient groups measured using computed tomography (CT) or cephalograms (CGs). This meta-analysis provides clinically useful information on ST morphology.

## 2. Materials and Methods

### 2.1. Search Strategy

For the sake of this meta-analysis, a systematic search involved all articles in which the morphology of the ST was evaluated. Major online databases such as PubMed, Scopus, Embase, Web of Science, and the Cochrane Library were searched through. The overall search process was conducted in three stages. (1) In the first step, all mentioned medical databases were searched using the following search terms: [(sella turcica) OR (turkish saddle) OR (hypophyseal fossa) OR (tuberculum sellae) OR (dorsum sellae)] AND [(anatomy) OR (topography) OR (variation) OR (structure) OR (size) OR (morphology) OR (width) OR (length) OR (type)]. No date, language, article type, or text availability conditions were applied. (2) Furthermore, the databases were searched through once again using another set of search phrases: (a) (sella turcica [Title/Abstract]) AND (anatomy [Title/Abstract]); (b) (sella turcica [Title/Abstract]) AND (topography [Title/Abstract]); (c) (sella turcica [Title/Abstract]) AND (variation [Title/Abstract]); (d) (sella turcica [Title/Abstract]) AND (structure [Title/Abstract]); (e) (sella turcica [Title/Abstract]) AND (size[Title/Abstract]); (f) (sella turcica[Title/Abstract]) AND (morphology[Title/Abstract]); (g) (sella turcica [Title/Abstract]) AND (width [Title/Abstract]); (h) (sella turcica [Title/Abstract]) AND (length [Title/Abstract]); (i) (sella turcica [Title/Abstract]) AND (type [Title/Abstract]). Additionally, each phrase was checked for differences in the structure of words (e.g., variation vs variations) and adapted if needed. (3) A manual search was also performed throughout all references from the initial submitted studies. The Preferred Reporting Items for Systematic Reviews and Meta-Analyses (PRISMA) guidelines were followed. Additionally, The Critical Appraisal Tool for Anatomical Meta-analysis (CATAM) and Anatomical Quality Assessment Tool (AQUA) were used to provide the highest-quality findings [16,17].

### 2.2. Eligibility Assessment and Data Extraction

**Figure 2:**
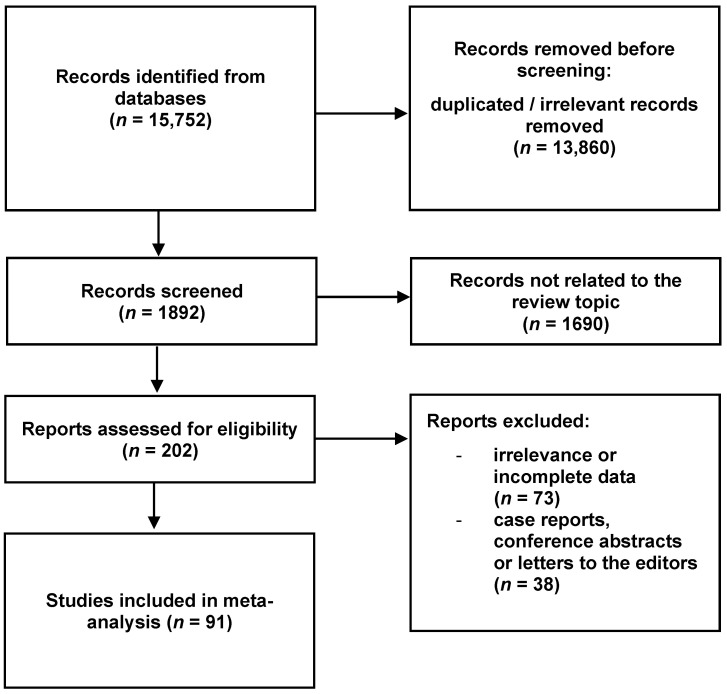
Flow diagram presenting the process of collecting data included in this meta-analysis.

The inclusion criteria were set as follows: original articles with extractable data on the anatomy of the ST. The exclusion criteria involved conference reports, case reports, case series, reviews, letters to the editor, and studies with no relevant or incompatible data. Two independent researchers performed a systematic search. A total of 15,752 articles were initially found. After the removal of duplicates and irrelevant reports, a total of 91 articles matched the required criteria and were taken into consideration in this meta-analysis [1,2,9,12,13,14,18,19,20,21,22,23,24,25,26,27,28,29,30,31,32,33,34,35,36,37,38,39,40,41,42,43,44,45,46,47,48,49,50,51,52,53,54,55,56,57,58,59,60,61,62,63,64,65,66,67,68,69,70,71,72,73,74,75,76,77,78,79,80,81,82,83,84,85,86,87,88,89,90,91,92,93,94,95,96,97,98,99,100,101,102]. The overall process of article collection is shown in Figure 2. Moreover, the characteristics of the submitted studies are presented in Table 1.

**Figure 3:**
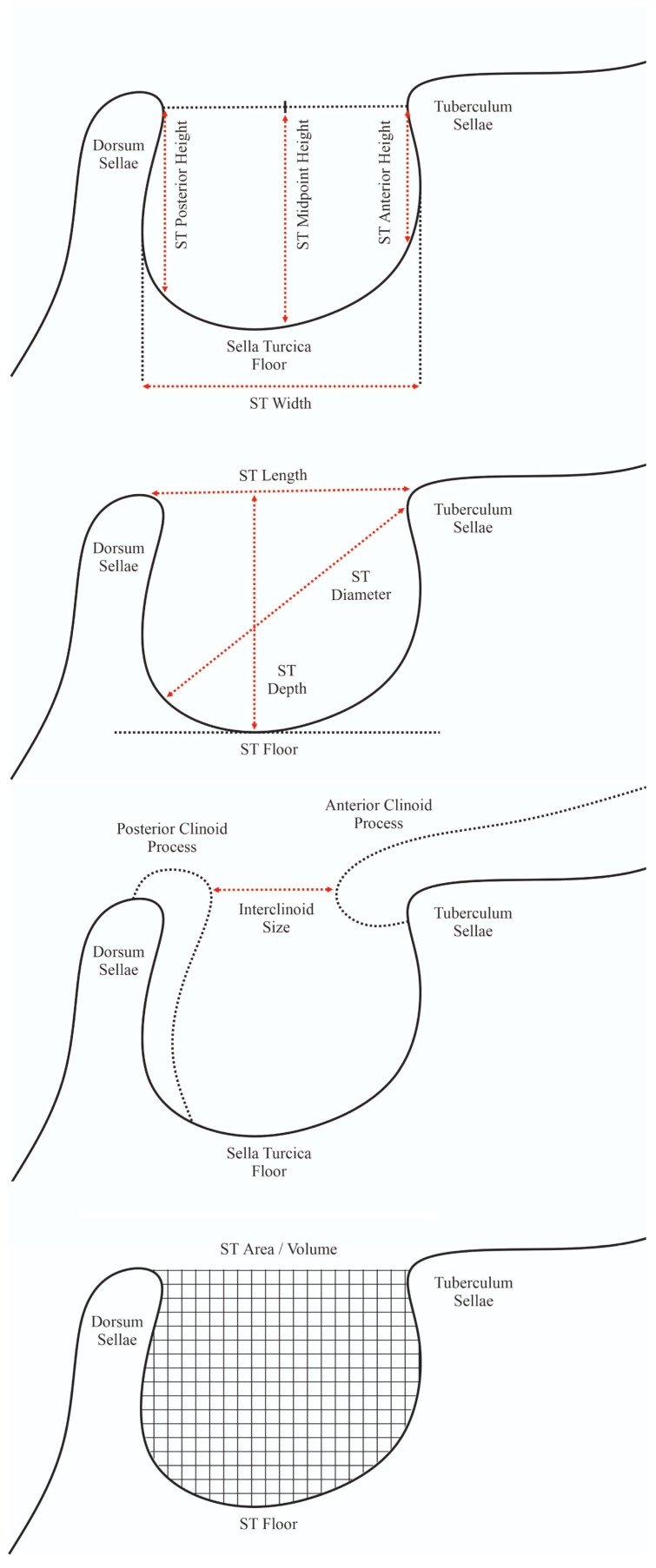
Illustration showing the dimensions of the sella turcica that were included in this meta-analysis.

Two independent researchers extracted data from qualified studies. Qualitative data were collected, such as the year of publication, country, and continent. Furthermore, quantitative data were gathered in several categories: (1) occurrence of specific anatomical variants of ST in the general population (according to various classifications); (2) height of ST; (3) length of ST; (4) width of ST; (5) diameter of ST; (6) area of ST; (7) depth of ST; (8) volume of ST; and (9) interclinoid size of ST (Figure 3). Any discrepancies between the studies identified by the two researchers were resolved by contacting the authors of the original studies whenever possible or by consensus with a third researcher.

### 2.3. Statistical Analysis

To perform this meta-analysis, STATISTICA version 13.1 software (StatSoft Inc., Tulsa, OK, USA), MetaXL version 5.3 software (EpiGear International Pty Ltd., Wilston, Queensland, Australia), and Comprehensive Meta-analysis version 4.0 software (Biostat Inc., Englewood, NJ, USA) were applied. A random effects model was used. The Chi-square test and the I-squared statistic were chosen to assess the heterogeneity among the studies [103,104]. The *p*-values and confidence intervals were used to determine the statistical significance between the studies. A *p*-value lower than 0.05 was considered statistically significant. The differences were considered statistically insignificant in the event of overlapping confidence intervals. I-squared statistics were interpreted as follows: values of 0–40% were considered as “might not be important”, values of 30–60% were considered as “might indicate moderate heterogeneity”, values of 50–90% were considered as “may indicate substantial heterogeneity”, and values of 75–100% were considered as “may indicate substantial heterogeneity”.

## 3. Results

This meta-analysis was finally based on the results of 18,364 patients. The prevalence of different types of ST was estimated using four classification methods.

The first classification method was general classification (Table 2). In this categorizing mode, the most prevalent ST type was the normal ST, with a pooled prevalence of 55.56% (95% CI: 49.32–61.71%). “Normal ST” was classified as ST without sella turcica bridges, irregularities of the posterior part of the dorsum sella, oblique anterior wall, the double contour of the floor, or a pyramidal shape of dorsum sellae. The sella turcica bridging is an anatomical variant resulting from the partial or complete fusion of the anterior and posterior clinoid processes. Among reports using this classification, the sellar bridges were observed in 11.34% (95% CI: 8.21–14.91%). The results mentioned above were based on a total of 5406 patients.

In the second classification method (distinguishing U-shaped sella turcica, J-shaped sella turcica, and flat-shaped sella turcica on the saggital sellae section; Table 2), the most prevalent ST type was the U-shaped ST, with a pooled prevalence established at 73.58% (95% CI: 54.24–89.36%). Furthermore, J-shaped ST was found in 16.91% (95% CI: 10.29–24.72%). Those results were based on a total of 1511 patients.

**Figure 4:**
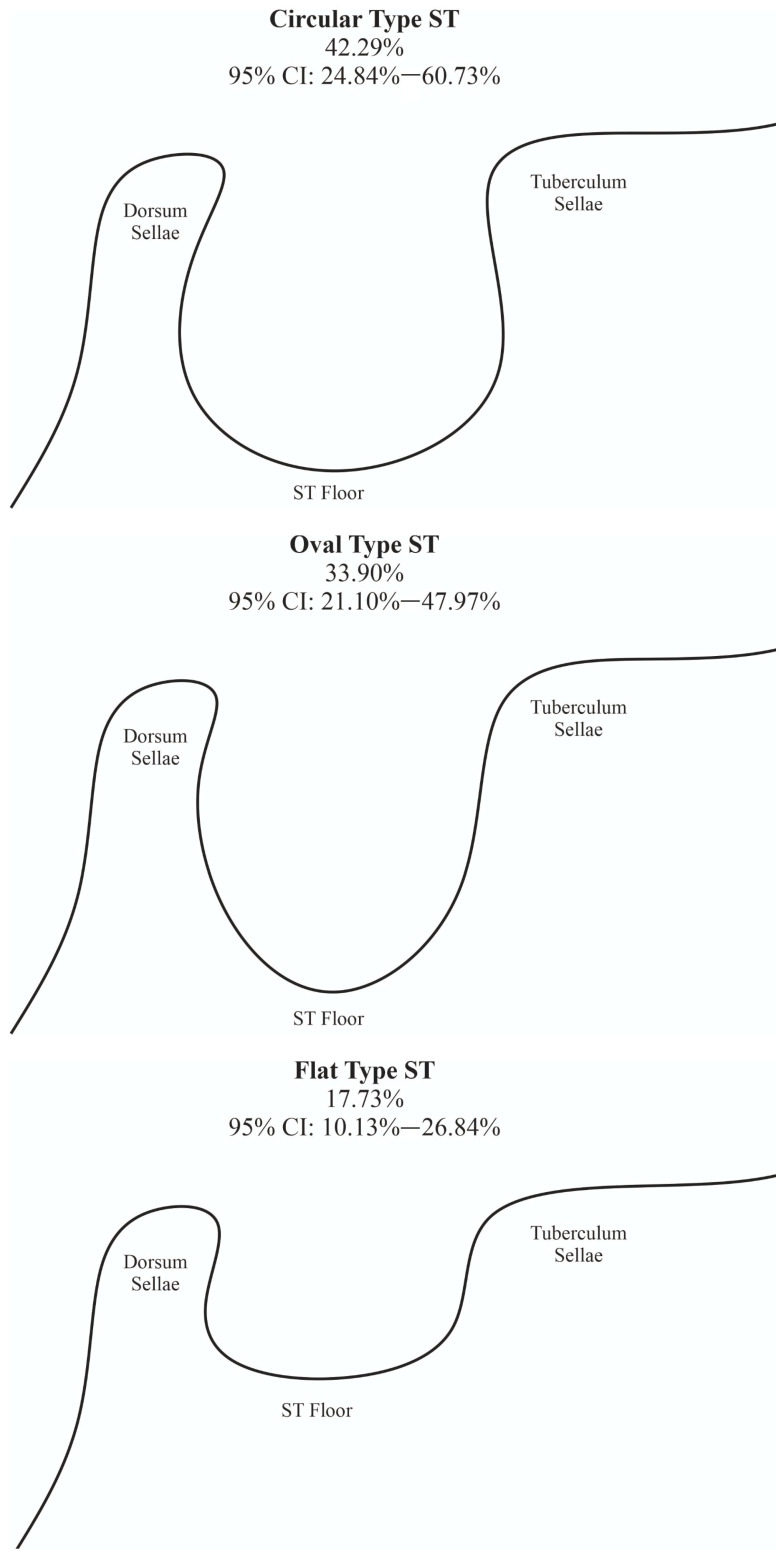
Illustration showing the oval, circular, and flat types of the sella turcica.

In the third classification method (recognizing circular type sella turcica, oval type sella turcica, and flat type sella turcica; Table 2 and Figure 4), the most prevalent ST type was the circular type of ST, with a pooled prevalence established at 42.29% (95% CI: 24.84–60.73%). Furthermore, the oval type of ST was present in 33.90% (95% CI: 21.10–47.97%). Those results were based on a total of 1191 patients.

In the fourth classification method, classifying types of sellar bridges (Table 2), the most common ST type was the type I, i.e., an ST without bridging, with a pooled prevalence of 55.64% (95% CI: 44.33–66.66%). Furthermore, type II, an ST with partial bridging, was found in 32.99% (95% CI: 24.22–42.39%). Those results were based on a total of 1791 patients. All of the results above and more detailed data regarding the types of ST are demonstrated in Table 2.

The main important ST morphometric characteristics are as follows. The overall anterior height of the ST was found to be 6.71 mm (SE = 0.52), whereas the midpoint and posterior heights of the ST were found to be 6.59 mm (SE = 0.13) and 6.93 mm (SE = 0.22), respectively. The detailed results regarding the height of the ST are presented in Table 3. The overall length of the ST was found to be 9.06 mm (SE = 0.15). In females, the ST length was 8.94 mm (SE = 0.22), whereas in males it was established as 9.19 mm (SE = 0.26). The overall width of the ST was 9.74 mm (SE = 0.31). In females, the width of the ST was 9.78 mm (SE = 0.39), whereas in males, it was estimated at 9.51 mm (SE = 0.40). All results regarding the ST length and width are shown in Table 4. The overall ST diameter was 11.15 mm (SE = 0.17). The overall ST area was 56.64 mm^2^ (SE = 8.79). Precise results regarding the diameter and area of the ST can be found in Table 5. The overall depth of the ST was found to be 8.00 mm (SE = 0.13). The overall volume of the ST was 845.80 mm^3^ (SE = 288.92). The overall interclinoid size of the ST was found to be 4.94 mm (SE = 0.50). Detailed results regarding those parameters are presented in Table 6.

## 4. Discussion

Three main classification systems that describe the morphology of the ST can be distinguished. The first one is derived from the study of Axelsson et al. [102], where seven ST types were distinguished. Cited authors applied the term “normal ST morphology” described by Björk and Skieller in 1983 [105]. That way, the classification was created, which divides the ST into normal ST, oblique anterior wall, sella turcica bridge (STB), double contour of the sella turcica floor, irregularities of the posterior part of DS, pyramidal shape of DS, and variants showing characteristics of more than one type. The prevalence of each type mentioned above varies wildly in the literature, with the normally shaped sella turcica present in most patients. For instance, Islam et al. [2] assessed the morphologic properties of the ST using CT images gathered from 166 patients. Those authors observed normal sella in 69.2% of subjects, while Isman et al. [1], using cone beam computed tomography (CBCT), described it in a remarkably lower percentage of patients, with only 49.8% having an ST of this kind. However, the current meta-analysis’s findings indicate that 55.56% of individuals have this form of ST. The prevalence and morphology of the second most common type varied between the studies. Islam et al. described irregularity (notching) in the posterior part of the dorsum sella in 16.2% of patients [2]. In comparison, Isman et al. reported a double contour of the sella turcica floor with a prevalence of 22.8% [1]. In contrast to those results, our study concluded that the sella turcica bridge (STB) was the second most common type, with a prevalence of 11.34%. Interestingly, in both studies mentioned above, the frequency of this morphologic type was on the lower end of the spectrum, with 0% and 3% of subjects presenting it, respectively [1,2].

It is crucial to note that some authors further divided ST using the classification by Leonardi et al. [106]. Those authors categorized ST into three groups based on the formation of the STB: no bridging (type I), partial bridging (type II), and complete calcification of the ST (type III) [106]. The assessment was based on comparing the length of ST and the anteroposterior greatest diameter. The ST length was measured as the distance between the tuberculum sellae and the tip of the dorsum sellae, and the anteroposterior greatest diameter was measured between the tuberculum sellae and the furthest point on the interior wall of the pituitary fossa. When the length was greater or equal to three-quarters of the diameter, ST was classified as type I; if this length was lesser, type II was assigned; and if there was a visible diaphragm sella (complete calcification of the interclinoid ligament), it was defined as type III. Notably, this method was only used in studies assessing ST with CGs. Interestingly, most studies reported type I with the highest prevalence, although the exact percentage varied across analyzed papers [45,55,87]. The results of the present meta-analysis align with the literature data, with 55.64% frequency for type I. It is also worth mentioning that the forming sellar bridges in the ossification stage were observed even during the fetal period (one observation of the fetal skull), suggesting that this type of ST formation may also have a developmental background, contrary to age-related calcifications occurring in some cases [107].

Another classification system that presents the morphological pattern of the ST is based on the resemblance of the letters U and J, as proposed by Ruiz et al. (2008) [74]. The U-shaped ST is distinguished when the sellar tubercle (tuberculum sellae) and the dorsum sellae (DS) are maintained at the same height. The J-shaped ST is described when the sellar tubercle is positioned inferior to the DS. The third type appearing in this classification, the flat-shaped (shallow) ST, is described when the depth of ST is minimal [66]. Hasan et al. [66] analyzed CT scans of 71 individuals and found that 50.7% had a U-shaped ST, 32.4% had a J-shaped ST, and 16.9% presented with a flat-shaped ST. However, a study by Muhammed et al. [54] concerning differences in ST morphology between Bosnian and Iraqi populations concluded that in both groups, the prevalence of U-shaped was much higher, with a prevalence of 86.7%. Accordingly, the frequency of other types was curtailed, with 12.2% and 9.4% possessing the J-shaped ST and 1.1% and 3.9% possessing the flat-shaped ST in both populations, respectively [54]. The results of our meta-analysis indicate that the distribution of each type of ST differs from the above papers. Nevertheless, the U-shaped ST is still the most prevalent, with 73.58% of individuals possessing it.

Another less frequently used classification system dating back to 1923 was provided by Gordon et al. [2,31,95,108]. This classification categorizes the ST as circular, oval, or flat-shaped. Most past research demonstrated the oval type as the most prevalent. However, in two separate articles conducted by Yasa Y. et al. [31,32], the most frequent types of ST were stated to be circular, seen in 65.3% and 69.5%. Our statistical analysis revealed a significantly lower percentage of individuals classified as having the circular type of ST, specifically 42.29%. Conversely, the oval type was observed in 33.9% of the subjects.

The morphometric properties of the ST have been widely discussed in the literature. The heights of ST were measured as a distance between the sella floor and certain structures perpendicular to the Frankfort horizontal plane [109]. This plane is described as a line connecting the left orbital and both Porion points, the most superiorly positioned points of each external auditory meatus [109,110]. To estimate the anterior height, ST was used as a landmark; to estimate the posterior height, the posterior clinoid process (PClin) was chosen; and to estimate the median height, a point midway between TS and PClin was used [22,28,83,88]. When analyzing the results obtained in our meta-analysis, both anterior and posterior ST heights varied considerably between the two radiological modalities, i.e., CT and CG. Both measurements were significantly lower in patients evaluated by CT; however, the standard error was much higher, suggesting that the sample of cases examined by this method was too small. Interestingly, the dimensions established in patients with a cleft lip and palate were decreased compared to the overall results.

Moreover, the results of the present meta-analysis show the ST length, measured as a distance from TS to PClin [22], to be the longest in the North American population and the shortest among Europeans. It is worth noting that the standard error was significantly higher in the former group, most likely because of the scarcity of studies. Hence, the results may not be fully representative of that population. The diameter of ST varied considerably in the literature, from 9.4 mm [69] to 14.24 mm [46]. Our meta-analysis concluded it to be 11.15 cm, without special deviations between Europeans and Asians or differences resulting from using various radiological methods. However, we noticed a slightly smaller diameter of ST in subjects with skeletal Class II compared to other skeletal classes. This observation was, to some extent, expected, looking at previous studies [26,39,78]. Nevertheless, some previous papers comparing measurements between different skeletal classes described the diameter of the ST as being higher in skeletal Class II compared to skeletal Class I or even found it to be the biggest among all skeletal classes [21]. On top of that, the mean ST diameter in patients with Down Syndrome was close to that of healthy individuals; however, further studies are needed to evaluate those calculations. Interestingly, the ST area, i.e., the area outlined by the sella contour and line joining TS and PClin [22,83], was calculated to be much smaller in patients with any type of cleft lip and palate compared to healthy individuals (43.12 mm^2^ vs. 56.64 mm^2^). In addition, the ST area was higher in Asians compared to the European population (62.32 mm^2^ vs. 56.29 mm^2^).

The mean ST depth varied significantly in the literature from 6.4 mm [39] to 10.87 mm [23]. The results of the present meta-analysis show that the overall depth of the ST is 8.00 mm, with minor differences between the analyzed groups. In addition, the ST depth was smallest in patients with skeletal Class II, which aligns with most studies [26,39,78]. The literature provides differences in ST volume ranging from 340.5 mm^3^, described by Halit et al. (2014), to 1428 mm^3^, described by Singhellakis et al. (1983) [37,75]. In our meta-analysis, the mean ST volume was 980.75 mm^3^. Interestingly, the overall calculated ST volume was smaller (845.8 mm^3^), but the standard deviation was high; hence the values varied greatly between individuals.

As the pituitary gland is located within the pituitary fossa and markedly influences the size of ST [12], any deviation in its measurements may reflect possible pathologies, even before they become clinically evident [83]. While CGs are not considered the method of choice for diagnosing pituitary tumors, the presented data can be used to avoid overlooking any incidental findings [83]. Therefore, up-to-date data concerning the ST’s morphology can help clinicians more accurately spot abnormalities in the PG size assessed using CGs and more sensitive methods, such as CT. Moreover, this knowledge is crucial for neurosurgeons performing surgery on the cranial base to choose the most suitable technique and spare damage to the pituitary gland and adjacent structures [49]. On top of that, more accurate calculations are also crucial in orthodontics, as ST is an essential anatomical determinant utilized to diagnose maxillofacial disharmonies such as palatally displaced canines or the congenital absence of the second mandibular premolar [12,55,106]. It can also help assess the results of orthodontic treatment [12]. The ST is also used as a landmark in lateral cephalometric analysis to assess the jaw relationship [49].

### Study Limitations

The present meta-analysis has several limitations. Due to the nature of the systematic review, the accuracy of the final results depends on the quality of the primary studies. A few limitations must be taken into account regarding this issue. First, the term population is often overused in research and refers to a sample that may only partially reflect the characteristics of the general population. It should also be considered that the samples used in the classification of U, J, and flat-shaped ST mainly consist of Bosnian, Chinese, Nepalese, and Iraqi individuals; therefore, the data should be related to other ethnicities with great caution. Most STs were also studied in Asia (*n* = 8791). Therefore, the overall results of this meta-analysis may be burdened, as they may reflect the anatomical features of Asian individuals rather than the global population.

Additionally, some analyses concerning ST sexual dimorphism or the discrepancies between the results obtained with different measurement methods (or various diagnostic modalities) were not performed due to insufficient literature data or the significant risk of possible results bias. Another limitation is that the article is based on a mix of radiological imaging rather than anatomical studies involving dry skulls or wet specimens. In addition, data on internal carotid artery anatomical variations or anomalies in the vicinity of sella turcica were not included in the study. As described by Björk and Skieller [105], “During growth, sella turcica increases in size by apposition on tuberculum sellae and by resorption at the posterior wall and at the floor”. Thus, the sella turcica’s age-related changes should also be considered when assessing the sella turcica contour morphology and comparing various examined samples. Regardless of limitations, our meta-analysis attempts to establish the detailed morphology of the ST based on the data from the currently available literature, which meets the requirements of evidence-based anatomy.

## 5. Conclusions

Four main classification methods of ST morphology can be distinguished in the available literature. Various morphometric characteristics of the ST may be applied in clinical practice to evaluate its shape, dimensions, and normal or pathological variants.

## Figures and Tables

**Table 1:** Characteristics of the studies included in this meta-analysis.

First Author	Year	Continent	Country	Method	*n*
Acevedo et al. [45]	2019	North America	USA	Computed Tomography and Cephalograms	185
Afzal and Fida [93]	2019	Asia	Pakistan	Cephalograms	180
Alkofide [85]	2008	Asia	Saudi Arabia	Cephalograms	285
Alkofide [86]	2007	Asia	Saudi Arabia	Cephalograms	180
AL-Mohana et al. [87]	2021	Asia	Singapore	Cephalograms	234
Alqahtani [84]	2019	Asia	Saudi Arabia	Dental Panoramic Tomography	98
Andredaki et al. [83]	2007	Europe	Greece	Cephalograms	184
Antonarakis et al. [82]	2020	Europe	Switzerland	Cephalograms	112
Antonarakisa et al. [101]	2022	Europe	Switzerland	Cephalograms	49
Arthisri et al. [63]	2021	Asia	India	Cephalograms	200
Axelsson [102]	2004	Europe	Denmark	Cephalograms	72
Baidas et al. [96]	2018	Asia	Saudi Arabia	Cephalograms	116
Canigur Bavbek and Arslan Avan [79]	2021	Europe	Turkey	Cephalograms	68
Canigur Bavbek and Dincer [80]	2014	Europe	Turkey	Cephalograms	152
Buyuk et al. [13]	2018	Asia	Japan	Cephalograms	410
Chaitanya and Chhaparwal [78]	2018	Asia	India	Cephalograms	480
Chou et al. [89]	2020	Asia	Taiwan	Computed Tomography	159
Dimario et al. [76]	1993	North America	USA	Cephalograms	58
Diri et al. [75]	2014	Europe	Turkey	Magnetic Resonance Imaging	96
Dixit et al. [19]	2017	Asia	Nepal	Cephalograms	473
Ekblom et al. [73]	2009	Europe	Finland	Computed Tomography	20
El-Sehly et al. [88]	2018	Africa	Egypt	Computed Tomography	215
Ferreri et al. [72]	1992	South America	Argentina	Cadavers	57
Gargi et al. [70]	2018	Asia	Singapore	Computed Tomography	100
Gibelli et al. [68]	2018	Europe	Italy	Computed Tomography	300
Gibelli et al. [69]	2015	Europe	Italy	Cephalograms	177
Goyenc et al. [67]	2008	Europe	Turkey	Radiographs	36
Gulsun et al. [97]	2020	Europe	France	Computed Tomography	100
Hasan et al. [65]	2019	Asia	Iraq	Computed Tomography	100
Hasan et al. [66]	2016	Asia	Iraq	Computed Tomography	71
Henriquez et al. [25]	2010	South America	Chile	Cephalograms	88
Ishikawa et al. [71]	1988	Asia	Japan	Computed Tomography	11
Islam et al. [2]	2017	Asia	Japann	Computed Tomography	166
Isman et al. [1]	2019	Europe	Turkey	Computed Tomography	200
Jankowski et al. [9]	2021	Europe	Poland	Cephalograms	206
Jones et al. [100]	2005	Europe	United Kingdom	Cephalograms	300
Kadam et al. [21]	2019	Asia	India	Cephalograms	90
Karaman et al. [81]	2018	Asia	Indonesia	Cephalograms	66
Kashio et al. [61]	2017	Asia	Japan	Cephalograms	232
Konwar et al. [20]	2016	Asia	India	Cephalograms	100
Korayem and AlKofide [60]	2014	Asia	Saudi Arabia	Cephalograms	120
Kucia et al. [59]	2014	Europe	Poland	Cephalograms	322
Kyung et al. [58]	2014	North America	USA	Magnetic Resonance Imaging	48
Lundberg et al. [57]	1975	North America	USA	Cephalograms	103
MacDonald et al. [94]	2022	North America	Canada	Cephalograms	1765
Magat and Ozcan Sener [49]	2018	Europe	Turkey	Cephalograms	362
Marcotty et al. [56]	2009	Europe	Germany	Cephalograms	400
Marşan and Öztaş [18]	2009	Europe	Turkey	Cephalograms	118
Mølsted et al. [98]	2009	Europe	Denmark	Cephalograms	105
Muhammed et al. [54]	2019	Asia	China	Cephalograms	540
Muhammed et al. [55]	2018	Asia	Japan	Cephalograms	360
Muhr et al. [53]	1981	Europe	Sweeden	Computed Tomography	205
Mustafa et al. [95]	2018	Europe	Italy	Cephalograms	509
Neha et al. [22]	2016	Asia	India	Cephalograms	110
Nerurkar et al. [77]	2022	Asia	India	Cephalograms	46
Neşat et al. [30]	2021	Europe	Turkey	Computed Tomography	188
Oon [52]	1963	Asia	Singapore	Cephalograms	260
Ortega-Balderas et al. [90]	2021	North America	Mexico	Computed Tomography	173
Otuyemi et al. [43]	2017	Africa	Nigeria	Cephalograms	117
Paknahad et al. [51]	2017	Asia	Iran	Computed Tomography	60
Arcos-Palomino and Ustrell [64]	2019	Europe	Spain	Cephalograms	150
Peker et al. [50]	2006	Europe	Turkey	Cadavers	80
Pigolkin et al. [24]	2018	Asia	Russia	Cadavers	86
Rai et al. [48]	2016	Asia	India	Cephalograms	32
Ruiz et al. [74]	2008	South America	Brasil	Computed Tomography	100
Russell and Kjar [47]	1999	Europe	Denmark	Radiography	78
Saokar et al. [62]	2022	Asia	India	Cephalograms	100
Sathyanarayana and Kailasam [26]	2012	Asia	India	Cephalograms	180
Sato and Endo [46]	2020	Asia	Japan	Cephalograms	166
Scribante et al. [44]	2017	Europe	Italy	Cephalograms	205
Shaha et al. [29]	2018	Asia	India	Computed Tomography	1650
Shaha et al. [27]	2017	Asia	India	Computed Tomography	200
Sherif et al. [42]	1989	Africa	Libya	Computed Tomography	74
Shrestha et al. [39]	2018	Asia	Nepal	Cephalograms	120
Silveira et al. [38]	2020	South America	Brasil	Computed Tomography	95
Singhellakis et al. [37]	1983	Europe	Greece	Radiography	883
Sinha et al. [36]	2019	Asia	Saudi Arabia	Cephalograms	300
Sobouti et al. [40]	2018	Asia	Iran	Cephalograms	105
Sobuti et al. [41]	2018	Asia	Iran	Radiography	105
Subasree and Dharman [23]	2019	Asia	India	Cephalograms	102
Sundareswaran and Nipun [35]	2015	Asia	India	Cephalograms	128
Surana et al. [14]	2022	Asia	India	Cephalograms	180
Taner et al. [92]	2018	Europe	Turkey	Computed Tomography	80
Tepedino et al. [91]	2015	Europe	Italy	Cephalograms	44
Trepedino et al. [12]	2019	Europe	Italy	Cephalograms	78
Turamanlar et al. [28]	2017	Europe	Turkey	Computed Tomography	101
Ugurlu et al. [34]	2019	Europe	Turkey	Computed Tomography	63
Valizadeh S. [99]	2015	Azja	Iran	Cephalograms	90
Yalcin [33]	2019	Europe	Turkey	Computed Tomography	136
Yasa et al. [31]	2017	Europe	Turkey	Computed Tomography	177
Yasa et al. [32]	2016	Europe	Turkey	Computed Tomography	139

**Table 2:** Results showing the pooled prevalence of each sella turcica type concerning different classification methods. LCI—lower confidence interval. HCI—higher confidence interval. Q—Cochran’s Q.

Category	*n*	Pooled Prevalence	LCI	HCI	Q	I^2^
Classification method I						
Normal sella turcica	5406	55.56%	49.32%	61.71%	698.43	95.13
Sella turcica bridge	5406	11.34%	8.21%	14.91%	781.78	94.50
Irregularities of the posterior part of the dorsum sella	5406	9.74%	7.53%	12.18%	206.74	84.52
Oblique anterior wall	5406	9.55%	7.30%	12.06%	221.36	85.54
Double contour of the floor	5406	6.89%	4.93%	9.15%	185.33	85.43
Pyramidal shape of dorsum sellae	5406	6.60%	5.12%	8.24%	129.76	75.34
More than one type	5406	1.10%	0.04%	3.14%	11.20	55.34
Classification method II						
U-shaped sella turcica	1511	73.58%	54.24%	89.36%	476.01	98.32
J-shaped sella turcica	1511	16.91%	10.29%	24.72%	113.50	92.95
Flat-shaped sella turcica	1511	6.89%	0.74%	17.23%	295.33	97.29
Classification method III						
Circular type sella turcica	1191	42.29%	24.84%	60.73%	178.13	97.19
Oval type sella turcica	1191	33.90%	21.10%	47.97%	108.81	95.40
Flat type sella turcica	1191	17.73%	10.13%	26.84%	63.78	92.16
Classification of the bridging type sella turcica						
Type I: no bridging	1791	55.64%	44.33%	66.66%	181.35	95.59
Type II: partial bridging	1791	32.99%	24.22%	42.39%	133.54	94.01
Type III: complete calcification	1791	10.27%	6.08%	15.37%	82.16	90.26

**Table 3:** Statistical results of this meta-analysis regarding mean height of the sella turcica (ST). CT—computed tomography.

Category	Mean [mm]	Standard Error	Variance	Lower Limit	Upper Limit	Z-Value	*p*-Value
Overall results							
ST anterior height	6.71	0.52	0.27	5.70	7.72	13.02	0.00
ST midpoint height	6.59	0.13	0.02	6.35	6.84	52.66	0.00
ST posterior height	6.93	0.22	0.05	6.50	7.35	31.97	0.00
Results established on CT							
ST anterior height	5.56	1.61	2.60	2.40	8.73	3.45	0.00
ST posterior height	6.46	0.54	0.29	5.40	7.52	11.93	0.00
Results established on cephalograms							
ST anterior height	7.24	0.22	0.05	6.81	7.68	32.69	0.00
ST midpoint height	6.59	0.13	0.02	6.35	6.84	52.66	0.00
ST posterior height	7.16	0.23	0.05	6.71	7.61	31.49	0.00
Results established on a European population							
ST anterior height	7.12	0.31	0.10	6.50	7.73	22.60	0.00
ST midpoint height	6.88	0.23	0.05	6.44	7.33	30.51	0.00
ST posterior height	6.89	0.23	0.05	6.45	7.33	30.60	0.00
Results established on an Asian population							
ST anterior height	7.29	0.55	0.30	6.21	8.36	13.30	0.00
ST posterior height	7.23	0.56	0.32	6.13	8.33	12.84	0.00
Results established in patients with any type of cleft lip and palate							
ST anterior height	6.10	0.13	0.02	5.85	6.35	48.08	0.00
ST midpoint height	6.10	0.10	0.01	5.91	6.29	62.51	0.00
ST posterior height	6.10	0.10	0.01	5.91	6.29	62.51	0.00

**Table 4:** Statistical results of this meta-analysis regarding mean length and width of the sella turcica (ST). CT—Computed Tomography. CG—Cephalograms.

Category	Mean [mm]	Standard Error	Variance	Lower Limit	Upper Limit	Z-Value	*p*-Value
Length							
Overall ST length	9.06	0.15	0.02	8.77	9.35	61.41	0.00
ST length (females)	8.94	0.22	0.05	8.50	9.38	40.03	0.00
ST length (males)	9.19	0.26	0.07	8.68	9.70	35.08	0.00
ST length (CT)	9.63	0.20	0.04	9.23	10.03	47.01	0.00
ST length (CG)	8.74	0.21	0.04	8.33	9.15	41.65	0.00
ST length (cadavers)	10.87	0.69	0.47	9.52	12.21	15.80	0.00
ST length (Europe)	8.91	0.31	0.10	8.30	9.53	28.42	0.00
ST length (Asia)	9.07	0.19	0.04	8.70	9.44	47.81	0.00
ST length (North America)	9.52	0.95	0.90	7.67	11.38	10.05	0.00
ST length (Africa)	9.09	0.54	0.29	8.03	10.15	16.79	0.00
ST length (any type of cleft lip and palate)	8.71	1.02	1.04	6.71	10.71	8.54	0.00
ST length (skeletal Class I)	7.92	0.84	0.70	6.28	9.56	9.48	0.00
ST length (skeletal Class II)	8.48	0.30	0.09	7.89	9.07	28.17	0.00
ST length (skeletal Class III)	8.72	0.45	0.21	7.83	9.61	19.17	0.00
ST length (patients with Down syndrome)	9.01	1.20	1.44	6.65	11.36	7.50	0.00
Width							
Overall ST width	9.74	0.31	0.09	9.13	10.34	31.66	0.00
ST width (females)	9.78	0.39	0.15	9.02	10.54	25.30	0.00
ST width (males)	9.51	0.40	0.16	8.74	10.29	24.01	0.00
ST width (CT)	10.35	0.24	0.06	9.88	10.81	43.47	0.00
ST width (CG)	9.13	0.28	0.08	8.59	9.67	33.10	0.00
ST width (cadavers)	11.99	0.50	0.25	11.01	12.97	23.98	0.00
ST width (Europe)	10.25	0.36	0.13	9.54	10.95	28.38	0.00
ST width (Asia)	8.89	0.37	0.14	8.16	9.62	23.89	0.00
ST width (any type of cleft lip and palate)	8.60	0.14	0.02	8.32	8.88	60.74	0.00
ST width (skeletal Class I)	9.34	1.29	1.67	6.81	11.87	7.24	0.00
ST width (skeletal Class II)	8.11	0.90	0.82	6.33	9.88	8.97	0.00
ST width (skeletal Class III)	8.51	0.17	0.03	8.18	8.84	50.75	0.00

**Table 5:** Statistical results of this meta-analysis regarding mean diameter (mm) and area (mm^2^) of the sella turcica (ST). CT—computed tomography. CG—cephalograms.

Category	Mean [mm/mm^2^]	Standard Error	Variance	Lower Limit	Upper Limit	Z-Value	*p*-Value
Diameter							
Overall ST diameter	11.15	0.17	0.03	10.82	11.48	66.69	0.00
ST diameter (CT)	11.21	0.19	0.04	10.84	11.58	59.18	0.00
ST diameter (CG)	11.05	0.20	0.04	10.66	11.44	55.52	0.00
ST diameter (Europe)	11.18	0.31	0.10	10.57	11.78	36.17	0.00
ST diameter (Asia)	11.09	0.18	0.03	10.74	11.43	62.86	0.00
ST diameter (any type of cleft lip and palate)	10.97	0.86	0.73	9.29	12.65	12.80	0.00
ST diameter (skeletal Class I)	11.38	0.48	0.23	10.45	12.32	23.86	0.00
ST diameter (skeletal Class II)	10.80	0.42	0.17	9.98	11.61	25.98	0.00
ST diameter (skeletal Class III)	11.42	0.42	0.18	10.59	12.24	27.03	0.00
ST diameter (patients with Down syndrome)	10.80	2.21	4.88	6.46	15.13	4.89	0.00
Area							
Overall ST area	56.64	8.79	77.34	39.41	73.88	6.44	0.00
ST area (CT)	53.40	8.79	77.31	36.17	70.63	6.07	0.00
ST area (CG)	55.73	9.35	87.41	37.40	74.05	5.96	0.00
ST area (Europe)	56.29	4.17	17.41	48.11	64.46	13.49	0.00
ST area (Asia)	62.32	7.49	56.11	47.64	77.00	8.32	0.00
ST area (any type of cleft lip and palate)	43.12	0.93	0.86	41.30	44.94	46.38	0.00

**Table 6:** Statistical results of this meta-analysis regarding mean depth (mm), volume (mm^3^), and interclinoid size (mm) of the sella turcica (ST). CT—computed tomography. CG—cephalograms.

Category	Mean [mm/mm^3^]	Standard Error	Variance	Lower Limit	Upper Limit	Z-Value	*p*-Value
Depth							
Overall ST depth	8.00	0.13	0.02	7.75	8.25	62.40	0.00
ST depth (CT)	8.23	0.21	0.04	7.82	8.64	39.60	0.00
ST depth (CG)	7.86	0.16	0.02	7.55	8.16	50.64	0.00
ST depth (Europe)	7.81	0.19	0.04	7.44	8.19	40.70	0.00
ST depth (Asia)	8.06	0.20	0.04	7.68	8.45	40.95	0.00
ST depth (North America)	7.91	0.80	0.64	6.34	9.48	9.89	0.00
ST depth (any type of cleft lip and palate)	7.52	0.45	0.20	6.64	8.39	16.84	0.00
ST depth (skeletal Class I)	7.92	0.38	0.15	7.17	8.67	20.61	0.00
ST depth (skeletal Class II)	7.46	0.30	0.09	6.88	8.04	25.14	0.00
ST depth (skeletal Class III)	7.91	0.25	0.06	7.42	8.40	31.44	0.00
Volume							
Overall ST volume	845.80	288.92	83,475.48	279.52	1412.07	2.93	0.00
ST volume (CT)	969.68	53.17	2827.24	865.47	1073.90	18.24	0.00
ST volume (CG)	891.94	307.95	94,836.28	288.36	1495.53	2.90	0.00
ST volume (cadavers)	671.33	78.20	6115.48	518.06	824.60	8.58	0.00
ST volume (Europe)	980.75	30.91	955.38	920.17	1041.33	31.73	0.00
Interclinoid size							
Overall ST interclinoid size	4.94	0.50	0.25	3.97	5.92	9.95	0.00
ST interclinoid size (CG)	4.94	0.50	0.25	3.97	5.92	9.95	0.00
ST interclinoid size (Europe)	4.66	0.68	0.46	3.34	5.98	6.90	0.00

## Data Availability

All data are included in the article.
